# Supplementary material for: Transperineal US-MRI Fusion-Guided Biopsy for the Detection of Clinical Significant Prostate Cancer: A Systematic Review and Meta-Analysis Comparing Cognitive and Software-Assisted Technique
Source: Cancers (Basel). 2023 Jun 30;15(13):3443. doi: 10.3390/cancers15133443 (PMC10341093; doi:10.3390/cancers15133443)
Supplement: Supplementary file 1 [file cancers-15-03443-s001.zip › cancers-2420527-supplementary.pdf]

Supplementary Figure S1. Risk of bias of the included study (Rob2): **A)** review authors' judgments about each risk of bias item presented as percentages across all included studies; **B)** review authors' judgments about each risk of bias item for each included study.

A)

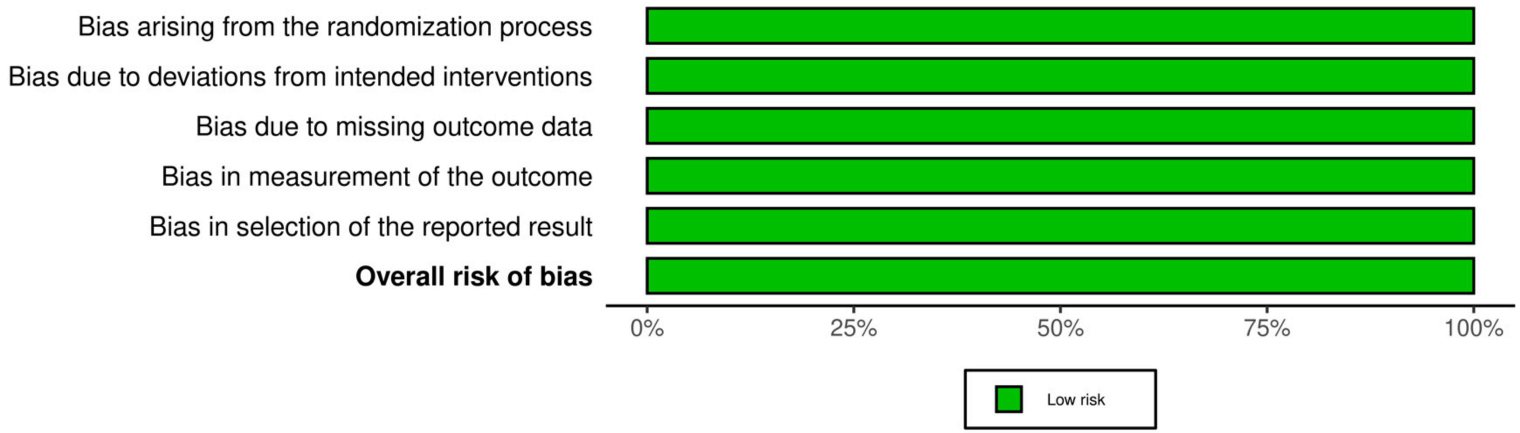

B)

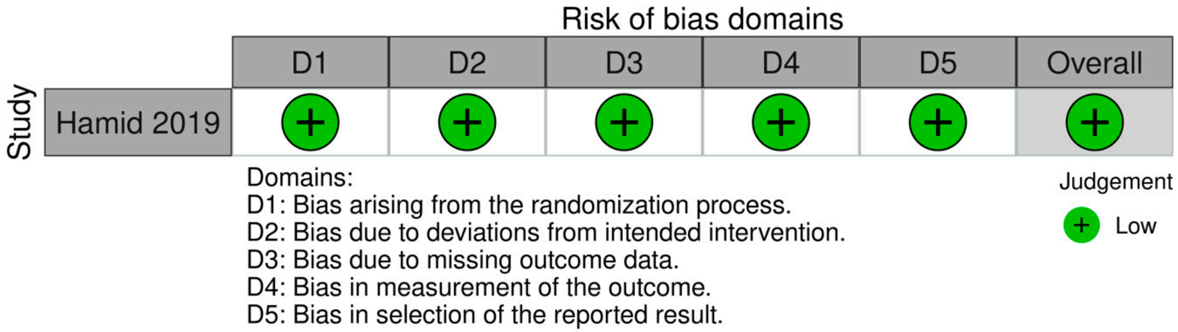

Supplementary Figure S2. Risk of bias of the included study (ROBINS-I): **A)** review authors' judgments about each risk of bias item presented as percentages across all included studies; **B)** review authors' judgments about each risk of bias item for each included study.

A)

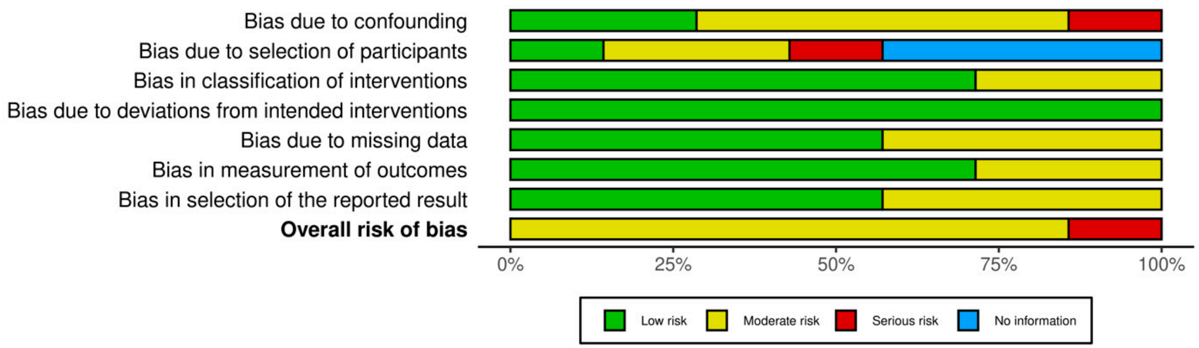

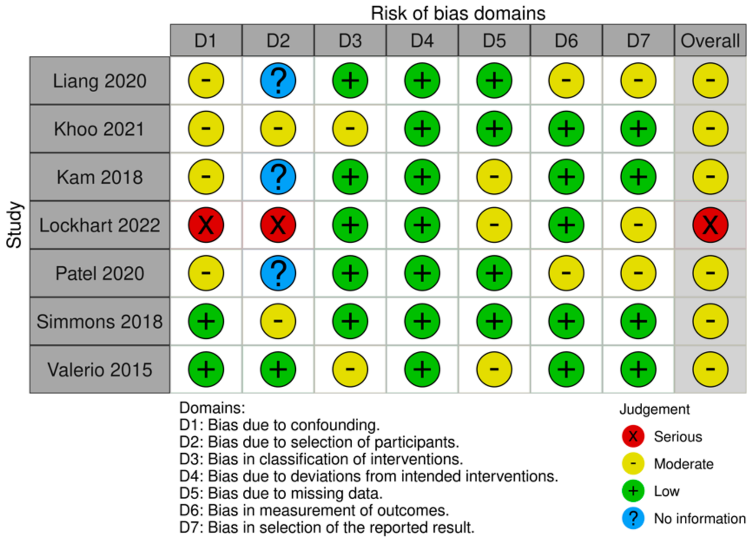

B)

Supplementary Figure S3. A) Funnel plot of meta-analysis of clinically significant prostate cancer detection rate in targeted lesions; **B)** Funnel plot of meta-analysis of clinically insignificant prostate cancer detection rate in targeted lesions.

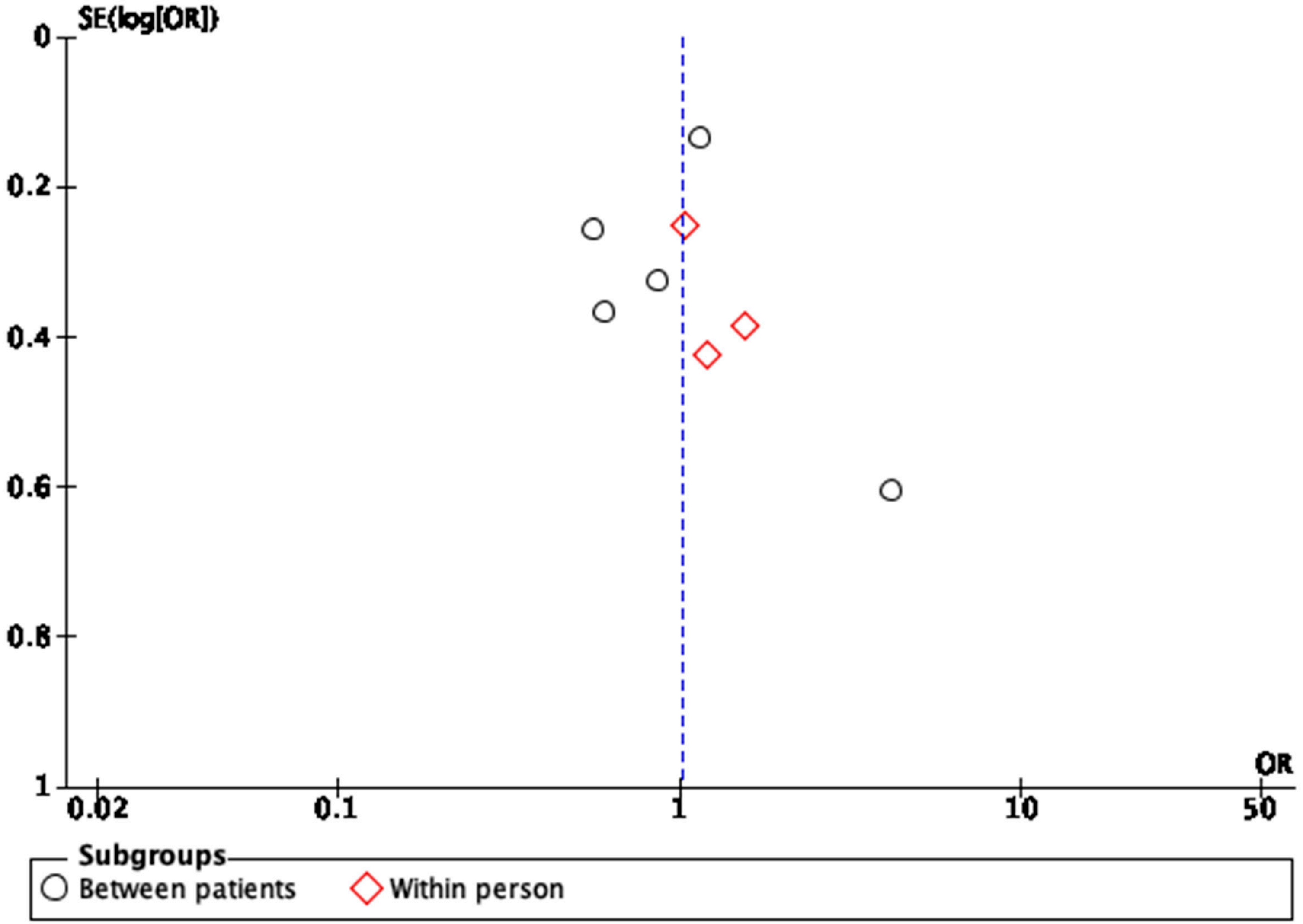

A)

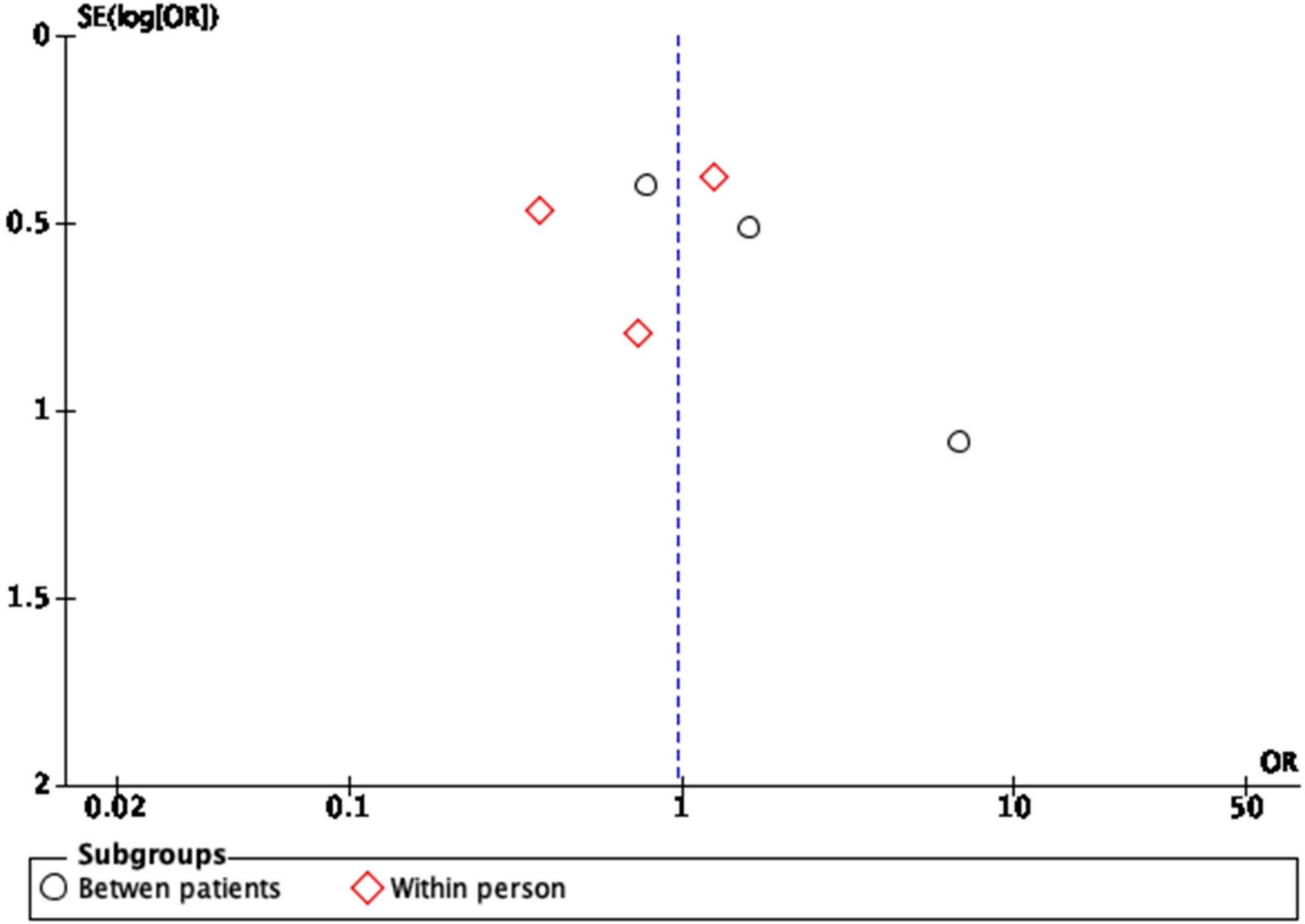
SE(log[OR])
0
0.5
1
1.5
2
OR
0.02
0.1
1
10
50
Subgroups
Betwen patients
Within person

B)
